# Public Involvement in a Systematic Review Project: Reporting Our Approach Using the ACTIVE Framework

**DOI:** 10.1111/hex.70323

**Published:** 2025-06-30

**Authors:** Carmel McGrath, Sarah R. Davies, Ifra Ali, Blessing Dick, Prakash Dewani, Clare E. French

**Affiliations:** ^1^ NIHR Health Protection Research Unit in Behavioural Science and Evaluation University of Bristol Bristol UK; ^2^ The National Institute for Health and Care Research Applied Research Collaboration West University Hospitals Bristol and Weston NHS Foundation Trust Bristol UK; ^3^ Faculty of Health and Applied Sciences, School of Health and Social Wellbeing University of West England Bristol UK; ^4^ NIHR Health Protection Research Unit in Vaccines and Immunisation, Faculty of Public Health and Policy, London School of Hygiene and Tropical Medicine London UK

**Keywords:** ACTIVE framework, feedback, public involvement, reciprocal learning, systematic reviews

## Abstract

**Introduction:**

Public involvement in research improves its relevance and accessibility to ensure it reflects the population it serves. However, there is a lack of information about how patients and the public are involved in shaping systematic reviews. There is also a need to address the lack of diversity in public involvement, particularly around the inclusion of underserved communities. This paper outlines the practical approaches taken to involve a diverse group of public contributors in a systematic review process and offers recommendations to support future practices.

**Methods:**

This paper uses the ACTIVE framework developed by Pollock et al. (2019) to structure our reflections on public involvement in an NIHR‐funded systematic review project on the effectiveness of interventions used to increase vaccination uptake.

**Results:**

We present the practical approaches used to recruit and engage a diverse group of public contributors in a systematic review project. They had continuous, hands‐on involvement in various aspects of the systematic review process. Our approach enabled the public involvement group to play an integral role in adapting and refining a coding framework, contributing to the development and refining of codes and categories used for the analysis.

**Discussion:**

Our paper highlights the importance of involving public contributors who have a range of experiences and backgrounds in the systematic review process. By investing time in relationship‐building, creating a safe environment and recruiting a diverse group of contributors, we gained richer insights that enhanced the coding framework. The complexity and nature of the methodology could make it challenging to identify where public contributors can make a real difference to research. Our experiences demonstrate that such involvement is possible and generates mutual benefits, including research that is better designed to reflect the diversity of the population.

**Conclusion:**

This paper outlines the steps undertaken to involve public contributors in the systematic review process. Key suggestions include investing time to build relationships, providing ongoing feedback, incorporating creative activities and developing strategies for disseminating research findings to wider audiences. These recommendations build on existing guidance and aim to support researchers in effectively involving public contributors in the systematic review process.

**Patient or Public Contribution:**

All public contributors working on the vaccination uptake project have been involved in reviewing this paper. Two public contributors P.D. and B.D. are also co‐authors and have provided input to the writing and review of this manuscript.

## 1 Introduction

The involvement of patients and the public in health research is an evolving practice and a requirement of national UK funders and ethics committees. As such, the literature and evidence base around patient and public involvement is growing, resulting in an increasing awareness of the value and benefits of knowledge exchange between researchers and individuals with relevant lived experience and experiential expertise.

However, patients and the public from underserved communities are under‐represented both as participants and public contributors in research [1, 2]. The term underserved communities in this paper refers to the UK National Institute for Health and Care Research (NIHR) definition: ‘A group that is less well represented in research than would be desirable from population prevalence and healthcare burden’ [2]. Not involving individuals from underserved communities in research means that research findings will be less likely to meet the needs of different communities. This is a problem as it is these communities who are more likely to be experiencing health inequalities and poorer health outcomes due to the disparities in access to care and treatment [1]. Therefore, we must continue to develop approaches to public involvement in research that are inclusive and accommodate our diverse communities so they can be involved in shaping research [2, 3, 4].

An area of research that has limited information regarding how patients and the public are involved is systematic reviews [5]. Systematic reviews are considered the highest level of evidence in evidence‐based medicine, using rigorous systematic processes to appraise and synthesise data from multiple studies addressing a particular research question and generating recommendations to inform policy and practice. Working with patients and the public can help ensure that review findings are translated and have relevance and impact for the wider population [6]. There are different terms used to describe the active involvement of patients and the public in research, including ‘citizen’, ‘public contributor’, ‘consumer’, ‘layperson’ or ‘service user’ [7, 8, 9, 10]. In this paper, we use the term ‘public contributors’ to describe members of the public contributing to research, as this is a well‐known term and is used by the NIHR.

Several systematic reviews have been published on the subject area of public involvement [11, 12, 13]. A scoping review of stakeholder involvement in systematic reviews by Pollock et al. in 2018 identified 291 papers reporting stakeholder engagement but only 30% of these reported involving patients and the public. Only 10% of the 291 papers provided detailed descriptions about the approach used for involvement [5].

The review highlighted the different ways in which people were recruited to be involved and the different ways people were involved, which were mainly through face‐to‐face meetings or Delphi surveys. Across the reviews included, there was a general lack of information about the financial incentives provided and evaluations. These knowledge gaps limit our understanding of public involvement practices in this area.

A later paper by Zhou et al., published in 2024, reported the frequency, determinants, stages and barriers to public involvement in systematic review projects [14]. The paper reports on public involvement in journals where there is a public involvement policy, in which all authors are required to complete a public involvement statement documenting how they included public contributors in their work. The British Medical Journal (BMJ) was the only journal that met these criteria, which put their policy in place in 2015 [14]. The paper included 217 reviews published between 2015 and 2022; only 56 included public involvement. Of the 161 reviews that did not report involving public contributors, 78.3% did not specify a reason and others highlighted potential barriers, including limitations in time (6.2%) and funding (11.8%) [14]. This proportion is surprisingly high given BMJ's policy.

In this paper, we aim to improve what is known about the involvement of public contributors in systematic reviews by outlining the practical approaches taken to involve a diverse group of public contributors in a systematic review process. We include reflections from the public contributors, highlight challenges encountered and based on our experiences, offer key recommendations (Table 3) for future practice in this area.

## 2 Project Background

This paper focuses on the involvement of public contributors in an NIHR systematic review project aiming to understand which interventions, or components of interventions, are most effective in increasing vaccine uptake in high‐ and upper‐middle‐income countries [15]. The review protocol is registered on the PROSPERO international prospective register of systematic reviews: https://www.crd.york.ac.uk/prospero/ [CRD42022369139]. The review includes studies on all vaccinations on the routine UK immunisation schedule across all age groups. The project is situated in a NIHR Health Protection Research Unit in Behavioural Science and Evaluation (HPRU BSE). This organisation is a partnership between the UK Health Security Agency (UKHSA) and the University of Bristol. The HPRU BSE is one of 14 HPRUs working across England. The research conducted by the HPRUs supports the UKHSA in protecting the public's health and minimising the health impact of emergencies.

Vaccines are powerful public health interventions helping to control infectious diseases and reduce morbidity and mortality, but rates of uptake in the United Kingdom are declining. The Covid‐19 pandemic highlighted that there are important differences in how communities respond to and engage with vaccination programmes. For example, underserved groups, including ethnic minority and socio‐economically deprived populations, have reportedly lower uptake of the Covid‐19 vaccine and other routine vaccines [16, 17, 18, 19, 20].

It was therefore crucial that we engaged a diverse group of public contributors who could provide a perspective that reflected the wider population to contextualise and provide further insight into the results that would be reported in the systematic review. In acknowledgement of the importance of public involvement in this project, a Senior Research Fellow in Public Involvement was costed in to lead on the public involvement and ensure that the approach to public involvement was appropriately designed and budgeted so that it could be embedded throughout the project.

## 3 ACTIVE Framework

In 2019, Pollock et al. published the ACTIVE framework which provides a useful guide for describing the approach to public involvement and covers the following areas: who is involved, how they are recruited, the mode and stage of involvement, and the level of control or influence they have in the review (see Table 1) [6]. The framework aims to provide guidance to researchers on how they can actively involve stakeholders in systematic reviews to ultimately enhance the quality, relevance and impact of the research findings. Here we use this framework to present the process of public involvement within our systematic review.

**Table 1:** The ACTIVE Framework constructs and categories developed by Pollock et al. (2019) [6].

Framework constructs	Categories
Who was involved?	– Patients, carers and/or their families – Patients, carers and/or their families + other stakeholders – Other stakeholders only
How were stakeholders recruited?	Open: – Fixed – Flexible Closed: – Invitation – Existing group – Purposive sampling
What was the mode of involvement?	Approach: – One‐time – Continuous – Combined (both one‐time and continuous) Methods: – Direct interaction – No direct interaction
At what stage in the review process did involvement occur?	(1) Develop a question. (2) Plan methods. (3) Write and publish protocol. (4) Develop search. (5) Run search. (6) Select studies. (7) Collect data. (8) Assess risk of bias. (9) Analyse data. (10) Interpret findings. (11) Write and publish review. (12) Knowledge translation and impact.
What was the level of involvement (at each stage)?	– Leading – Controlling – Influencing – Contributing – Receiving

## 4 Capturing Our Reflections

The information reported in this paper has been captured through discussions with members of the project team (C.F., S.D., C.M. and I.A.) who met every 2 months to discuss the approach to public involvement and through the reflective discussion held with the public contributors working on this project (S.S., N.G., F.O., M.F., S.B., L.B., N.C., M.O., C.L. and R.H.) (see ‘Reflecting on our approach to public involvement’ section).

Several meetings were held with the core authorship team (including the researchers [C.M., C.F., S.D. and I.A.] and public contributors [P.D. and B.D.]) to discuss the structure and content of the paper.

This section of the paper details the approach taken to involve public contributors in the systematic review project using the components within the ACTIVE framework (Table 1).

### 4.1 Who Was Involved?

The ACTIVE framework provides a simplified categorisation to differentiate the involvement of multiple stakeholders. These groups include patients, carers and/or family members and/or any other identified stakeholders [6].

For this project, we refer to the category of people involved as the ‘public’, given that the project topic—interventions to increase vaccine uptake across all age groups—is of relevance to the general population at large.

Given the variation in the uptake of vaccinations according to a range of characteristics, for example, socio‐economic status, ethnicity and cultural beliefs, the project team felt it was important to include people with a range of experiences and perspectives on vaccination. This included inviting people to join the group who might be hesitant to receive vaccinations. It was essential for the individuals involved to be a diverse group that reflects the wider population in terms of age, socio‐economic status, education, gender, ethnicity and religion. The team also wanted to ensure opportunities for joint learning; the people involved did not have to have prior experience in public involvement, vaccination topics or systematic reviews. Our approach to recruitment was successful, and 12 public contributors were recruited. Of the seven who responded to the demographic survey, the majority (57%) were aged 50 or older. The ethnic diversity of the group included 42.9% identifying as Black or Black British‐African, 28.6% as Black or Black British‐Any other Black background, with smaller numbers identifying as Black or Black British‐Caribbean (14.3%), Asian or Asian British‐Chinese (14.3%), Asian or Asian British‐Indian (14.3%), and White‐British (14.3%). 42.9% of members reported having a long‐standing illness. 42.9% of respondents were happy to receive recommended vaccines, with 42.9% of respondents expressing hesitancy, citing ‘general hesitation’ or ‘depending on the vaccine’. Further information on the self‐reported demographics of the group can be found in Appendix 1.

### 4.2 How Were Stakeholders Recruited?

The framework outlines two approaches for recruiting public contributors: ‘open’ and ‘closed’ recruitment. Open recruitment allows anyone to apply for the opportunity to be involved. Closed recruitment is targeted and means that people are usually recruited through existing groups or have already worked with the team and are recruited as they have a specific experience or expertise relevant to the research topic area [6].

We recruited public contributors by building on our existing relationships with communities and through our colleagues' connections. This closed approach to recruitment ensured that the people involved had a range of diverse backgrounds and experiences relevant to the topic area and an interest in the project.

In academia and research, funding deadlines can make it challenging to sustain relationships beyond project end dates. However, working with public contributors from previous projects enabled the team members to develop and build on existing relationships and trust with individuals, which was important to the research team. Furthermore, this continuity provided additional opportunities for public contributors to engage in research topics of interest to them [21, 22].

However, we also recognised that the initial group membership, which was drawn from an existing public involvement group, did not cover all the different groups we were interested in, so we also approached three additional public contributors. One public contributor was recruited through another public contributor. The other two public contributors were recruited through a colleague working in public involvement. No prior knowledge of the research topic area or systematic reviews was required before public contributors started the project. No initial training was given before their involvement, as the project team wanted to ensure the training would be relevant and designed to meet any knowledge gaps or learning needs.

### 4.3 Mode of Involvement

The ACTIVE framework describes the mode of involvement in terms of how people are engaged throughout the systematic review process. This can be either ‘one‐off’, where individuals are involved at a specific stage, or continuous, where the same people are engaged throughout the entire process. The framework further categorises involvement into three approaches: partnership, multiple‐time closed events and hands‐on approaches [6].

The methods of involvement described include direct interaction between stakeholders and the review team through face‐to‐face or virtual meetings and/or indirect methods, where involvement may occur through activities such as an electronic Delphi survey.

For this project, our approach aligned with the continuous, hands‐on approach. From the outset, the project team met with the public involvement group to discuss the research questions and how public involvement should be integrated throughout the process. It was collectively agreed that half the group would meet every 4 months (with six public contributors alternating at each meeting), and every 6 months, all twelve public contributors would meet. During these 6‐monthly meetings, public contributors also received training based on their expressed needs.

Additionally, two public contributors attended 6‐monthly project team meetings to share public involvement‐related updates with the wider project team members. Ahead of these 6‐monthly meetings, the public contributors were supported by the public involvement lead and a couple of key members of the project team to prepare for the meetings. A pre‐meeting was held with the public contributors to ensure they understood what the wider team meeting would involve, to review and contribute to materials beforehand, and to offer them opportunities to ask any questions ahead of the meeting.

Public involvement meetings were held in person (face‐to‐face) with the option for online participation. One public contributor (B.D.) and one member of the research project team (I.A.) consistently joined online. This did not limit the involvement of the public contributor as they attended every meeting, participated in the wider project team meetings and co‐authored this paper.

During meetings, the public contributor and the project team member joined on a laptop. For the breakout discussions, the public contributor, team member and public involvement lead would have a discussion as part of a smaller group. For the larger group discussions, the public involvement lead would take the laptop around to each table to ensure that the people online could hear the group discussions.

For those attending in person, meeting locations were chosen based on the group's preferences to ensure accessibility and comfort. All public contributors were reimbursed for their travel and compensated for their time according to the NIHR payment guidance for public involvement. Refreshments, including a selection of cakes, fruit and snacks based on the contributors' preferences, were provided. Time was always allocated for socialising and eating, contributing to a safe and welcoming environment. After the initial introductory meeting, the group discussed key considerations for working together (see Appendix 2). These considerations included ‘allowing others to talk one at a time, respecting everyone's comments and suggestions and asking questions throughout’. By creating these key considerations, it ensured that there were shared expectations about how we would work together.

Each meeting followed a specific format. The meetings began with a recap of the project and its aims, and the group were encouraged to ask any questions at this point. Following the recap, we moved to working in smaller groups for discussions and to complete activities. The small group activities were always followed by a whole‐group discussion, ensuring the smaller groups (which would include 4–5 public contributors per group) could provide feedback to the whole group on key areas discussed. The final part of the meetings included a summary of the day and plans for the next steps.

After each meeting, public contributors were asked to provide feedback, which was used to shape the following workshops. The feedback was based on responses to the following questions, which were developed by Wood (2021) [23]:

Head: What did you learn?

Heart: What did you love?

Hands: What will you take away?

Bin: What should we do differently?

### 4.4 Stage of Involvement

The framework describes the different stages of a systematic review process where involvement can occur, including developing the question, planning the methods and protocol, developing and running the searches, collecting and analysing data, interpreting findings, and publishing the review and in knowledge translation and impact.

To conduct the analyses, we needed to iteratively develop and refine a coding framework that enabled us to code important features of the content and delivery of interventions to increase vaccine uptake (e.g., who delivered in the intervention, mode of intervention delivery and intervention intensity). The public involvement group contributed to the development and refinement of codes and categories used for the analysis.

In this project, involvement has featured heavily in the coding and analysis phase of the process, which has influenced the direction of the systematic review. The public involvement group contributed to the development and refinement of codes and categories used for the analysis. In Table 2, we provide further information about the activities undertaken during each of our workshops to date. All workshops were 2–3 h in duration and held in community venues.

**Table 2:** A summary of our public involvement meetings and content.

Workshop number and date of workshop	Content of the workshop	Number of public contributors attending
Workshop 1, November 2022	This was the first workshop and involved the group getting to know each other, introducing the project and discussing how people would like to be involved moving forward. As a first activity, we asked the public contributors to place somewhere of importance to them on a map of the world (see Appendix 3). When introducing ourselves, we spoke about why this place was important. We also discussed the role descriptors (see Appendix 4) created for the public contributors and also talked about when our next meeting would occur.	Nine joined in person (we recruited two public contributors after this meeting). One public contributor and one project team member joined online There was full attendance at this meeting (10/10 public contributors attended).
Workshop 2, February 2023	During this workshop, we discussed aspects of the ‘5A's taxonomy’ for the determinants of vaccine uptake that was going to be used as the basis for developing a bespoke intervention coding framework to code the interventions used in the studies included in the review [24]. In small groups, we discussed factors that might need to be considered within the framework domains. For example, a theme emerging from the discussions was the importance of trust (e.g., is information about vaccination coming from a trusted source?). The input from the group fed directly into the coding of who delivered the intervention. These conversations with the group were important for highlighting factors that were particularly important to the group that the researchers had not considered, but might be detailed within the studies and could be coded within the categories. The discussions were also helpful for determining the suitability of this framework for the purpose of this review.	Five public contributors joined in person. One public contributor and one project team member joined online. There was full attendance at this meeting (6/6 public contributors attended)
Workshop 3, May 2023	We worked in small groups and discussed how we would individually code the intensity of different example interventions (each group had a different intervention) that were provided by the research team. We used flip chart sheets to ensure that everyone could be involved in the discussions and, at the same time, could note down any thoughts or ideas. After this exercise, we rejoined as a large group and talked through our perceptions of the intensity of the interventions. This was a useful discussion for highlighting to the team what level of intensity might be too much/or considered acceptable by the public. After this exercise, the public contributors received training from two external staff members (Dr. Andy Gibson and Dr. Abby Sabey) on the topic of understanding research evidence, which included systematic reviews. This training lasted for 1 h and was attended by all 12 public contributors. The session covered an introduction to the following areas: Understanding what research evidence is, factors that influence how we interpret information, why we need to use different research methods to understand different research questions and the stages involved in a systematic review process. This training was identified by the public contributors as training they would value to enhance their involvement and experience working on this project.	Eleven public contributors joined in person One public contributor and one project team member joined online. There was full attendance at this meeting (12/12 public contributors attended)
Workshop 4, October 2023	The activity for this workshop involved breaking into small groups, and each group were asked to group examples of who delivered the interventions into broader categories. Participants were free to choose as many broad categories as they felt they needed and name them in the ways they thought appropriate. This activity was designed to create smaller categories for coding the data. Once the activity had been completed in small groups, everyone rejoined as a larger group and discussed how they had categorised the examples. Although the public contributors were sitting in different groups, there was a clear consensus about how the data should be categorised, resulting in who delivered the intervention being coded as either ‘Trusted community member’ or ‘Healthcare practitioner’ in the coding framework (see Appendix 5).	Five public contributors joined in person. One public contributor and one project team member joined online. There was full attendance at this meeting (6/6 public contributors attended)
Workshop 5, February 2024	The public contributors received training from an external colleague on the topic: ‘An Introduction to Public Health Research’. This training was delivered by Dr. Andy Gibson and lasted for 1 h. The training covered the following areas: What is public health, definitions of public health, background and history of public health and the difference between health improvement and health protection with examples. All twelve public contributors attended the session. This training was requested by the public contributors, and after the content had been delivered, there was time for discussion and opportunities to ask questions. For the activity, the public contributors split into smaller groups and were asked to look at statements, which were excerpts from the data and described what people were told during some of the vaccine uptake interventions. The public contributors were then asked to categorise each statement and determine if it was education/information/motivational messaging and support intervention. This exercise was valuable as it allowed the project team to explore what the public contributors thought of as information and what was needed for it to be classed as education. It also highlighted that there may be several overlaps when they code into these different categories.	Eleven public contributors joined in person One public contributor and one project team member joined online. There was full attendance at this meeting (12/12 public contributors attended)
Workshop 6, July 204	The main focus of this meeting was to provide a space for the public contributors to reflect on the approach to public involvement on the project so far and to discuss what worked well and what could be improved.	Nine public contributors attended in person One public contributor and one team member joined online 10 out of 12 public contributors joined this session, with two unable to attend.

Two examples of how the public contributors have directly shaped the coding framework are included below:

1. When discussing offering incentives in the form of payment for having a vaccination, public contributors highlighted that this may cause hesitancy instead of encouraging uptake. They felt that generally people would understand why vaccinations were important for their overall health and well‐being, and therefore offering incentives such as money might raise concerns about why they were being ‘enticed’ with payment. As a result of this discussion, a separate category was created in the coding framework to capture studies that included offering monetary incentives for a vaccination and a separate category for offering reimbursement for travel.
2. The group emphasised the importance of information about vaccinations being delivered by a trusted person and where they are offered could make a difference. For example, the public contributors suggested that some communities may feel more comfortable engaging in a vaccination clinic that was held in a place of worship. They also reported that some people may trust individuals such as community leaders who share information about vaccinations with them. Following these discussions, categories were developed within the framework to capture information from the studies about who was delivering the intervention, in recognition that this could impact uptake of the intervention from different communities.

### 4.5 Level of Involvement

The ACTIVE framework provides a ‘continuum of involvement’ that suggests the extent to which public contributors can influence the project. The continuum ranges from ‘leading’, ‘controlling’ and ‘influencing’ to ‘contributing’ and ‘receiving’, and the framework provides an accompanying description next to each level [6].

The level of involvement from the public contributors on this project aligns with the contributing, influencing and receiving definitions provided within the framework [6]. The range of activities and involvement reported in this section highlights that within one stage of the systematic review process, there are multiple components and opportunities for public contributors to be actively involved. The public contributors also made the decisions around their involvement, including what training they wanted to receive, where the meetings should be held and how long the workshops should be.

## 5 Reflecting Our Approach to Public Involvement—The Public Contributors' Perspective

To capture the reflections from the public contributors involved in the project, a 2‐h meeting was held to discuss our approach to public involvement and identify areas for improvement. The discussions were audio‐recorded with permission. Ten public contributors attended this meeting, and the following sections summarise the discussions from the group, which were centred around the following four questions:

1. Have you had a voice in shaping the project?
2. Did the activities make you feel involved in the project?
3. Anything you particularly enjoyed or found interesting?
4. What worked well and what could be improved?

Before this meeting, the researchers (C.F., I.A. and S.D.) and public involvement Lead (C.M.) met with a public contributor (B.D.) to develop and discuss the questions to ensure they were suitable for this reflective conversation.

**Q1. Voice in Shaping the Project**

Three main areas were discussed in relation to whether people felt they had a voice in shaping the project: (i) group diversity, (ii) feeling safe and comfortable and (iii) feedback.

### 5.1 Group Diversity

When discussing group diversity, the public contributors noted the benefits of having a diverse group and recognised how the differences in views encouraged broader thinking and understanding of the same issue. These differences in views were considered important as they challenged the researchers and their own individual perspectives. It was felt that the group represented a good cross‐section of the community but could be even more diverse. In future public involvement groups, it was suggested that people should be invited to speak in their mother tongue, with the support of interpreters. This would encourage a richness of perspectives and insights that we may otherwise miss. The group also spoke about the benefits of having such a diverse group who were linked to different communities and, because of their involvement in this project, felt confident they had an important voice in bridging the gap between research and their communities.

### 5.2 Feeling Safe and Comfortable

Group members felt comfortable during the meetings, enabling them to openly speak their mind and share their honest views with the research team. They reported feeling that when sharing their perspectives, whether similar or different to the group, these views were always respected by the group. Group members also expressed that they felt supported by the project team members to speak when feeling less confident.

Communication in and between meetings enabled the public contributors to feel confident in knowing the planned next steps, and they expressed that they felt guided and supported throughout the project. The public contributors who joined the project at a later stage reported that the communication they received had helped to familiarise them with the project, and as a result, they could still contribute meaningfully to the project and ongoing discussions.

### 5.3 Feedback

The flexibility of meetings was valued, and the group could see how their suggested changes were incorporated into our future sessions. This response from the team contributed to building the confidence of the public contributors as they knew they were being listened to and that the meetings would be designed to meet their needs and preferences. They recognised how the researchers' open minds meant that meetings were tailored to the public contributors' needs. As a result, they felt they were on an equal footing with the research team and that the meetings and discussions were not dictated by the researchers' agendas. The group suggested that any researcher who was planning public involvement should include adequate budgets to be flexible and to ensure that the public involvement plans are influenced by the public contributors as the project developed.

**Q2. Feeling of involvement**

In response to whether the activities undertaken made people feel involved in the project, the following areas were discussed: (i) activity content and structure and (ii) positive impacts of the group dynamics.

### 5.4 Activity Content and Structure

The duration of group meetings varied. Some members of the group felt that the length of 2 h was a good balance of efficiency and engagement, meaning that people would not be fatigued but also allowed for in‐depth discussions. However, some members felt that a 3‐h session offered a more relaxed pace and allowed people to ease into the discussion. The group commented on the importance of time management and how this was essential to avoid rushing through discussions at the end of the meeting. They commented on the benefits of having a simple, structured approach to meetings and how this made it easy for everyone to contribute. The group also felt that outlining the aims of the meeting was important as it built trust and improved the public contributors' confidence in participating in meetings, as they were aware of what they were contributing to and how they could contribute.

The structure of the meeting was also recognised for creating inclusive spaces. The blend of small group and larger group discussions meant that people who were less comfortable speaking in larger groups could still contribute to the discussions, and the wider group discussions were considered to facilitate the diversity of perspectives in the room, generating new ideas and encouraging others to share their experiences and perspectives.

### 5.5 Positive Impact of Group Dynamics

The environment of the group meetings was described as non‐hierarchical, meaning that people felt they could share their perspectives freely and that their views were important and listened to.

One public contributor, before their involvement, thought that research was only for people who had a master's degree or a PhD. As a result of their involvement in this and other projects, they saw the value of their expertise and lived experience and how this perspective could positively impact the project.

The group expressed that they enjoyed hearing about people's different cultures and beliefs as part of discussions and the different points of view. Some felt that the differences in perspectives helped to challenge the thinking about the research but also deepened their own understanding.

One public contributor remarked that ‘it's the people that make the project’ and suggested that at times, researchers can be very focused on achieving project goals, rather than building relationships and getting to know the people in the room. It was considered important that researchers should invest time in building relationships with the people involved, and it was believed that this was an important priority for the research team working on this systematic review project. The group members reported that they appreciated that the research team had built in time during meetings for socialising and for ensuring that there were short‐term paybacks provided by the research team in the form of reimbursement and training opportunities.

**Q3. Enjoyment and interest**

When reflecting on what aspects of involvement people particularly enjoyed or found interesting, the following areas were discussed (i) personal growth and confidence, (ii) valued opportunities for learning and collaboration and (iii) awareness and interest in broader research.

### 5.6 Personal Growth and Confidence

When discussing why people enjoyed their involvement, they suggested that when starting new projects, particularly those which are more methodological in nature, it can be challenging to see how involving patients and the public can make a difference. Some commented that research itself can be seen as quite ‘dry’ and therefore difficult to know how it could translate into engaging activities that generate interesting insights and discussions with public contributors. However, for this project, because the group members had spent time with the research team and built relationships with them, they reported that their confidence had grown, and they understood how they could be involved and make a difference to the project. Others commented that they were inspired by watching the confidence in their peers grow throughout the duration of the project. For some, involvement in this project had been empowering, and as a result, they were confident in sharing information about their involvement with their family and friends.

### 5.7 Valued Opportunities for Learning and Collaboration

Members of the group valued the experience of being involved and commented that their experience had surpassed their expectations. For some, involvement in this project was of personal interest due to their professional backgrounds. For example, one member has a nursing background and was interested in the topic of vaccination. The group also appreciated the learning opportunities, such as the introduction to public health and systematic review training. They also identified gaps in their knowledge and requested training on how to write an academic paper and were interested in the process of preparing and submitting a manuscript for an academic journal.

### 5.8 Awareness and Interest in Broader Research

As a result of the training provided, the public contributors reported that they had a better understanding of how health projects work and could appreciate how much research goes on behind the scenes and how long it can take to produce a service or intervention. Their involvement in research meant they were more vigilant in different parts of their life, for example, one group member reported that they would now actively look at screens in GP surgeries with updates and key messages. They also noticed gaps in services and treatments, and public health messaging, and noted how more work needs to be done to ensure it reflects the diversity of our population. For example, the group member noticed that when advertising the whooping cough vaccination, there were no pictures of Black mothers with their babies.

**Q4. What worked well and areas for improvement?**

In terms of what worked well and what could be improved, the following areas were discussed: (i) session structure and group dynamics, (ii) communication and inclusivity, and (iii) diversity of views and representation.

### 5.9 Session Structure

As mentioned in previous sections, the group members considered the meeting structure of having smaller group discussions and then joining as a larger group to outline key points was effective in making sure everyone's voices were heard.

The characteristics of the researchers were considered by the group to be excellent as the group felt that they were friendly and encouraging in their approach. Members of the group also felt a personal investment in the project, as they felt important to the project and the project felt important to them.

While the group members felt that the table group discussions were engaging, a suggestion was made that the research team could consider incorporating interactive creative activities during the meetings, such as role‐play. It was suggested that including this interactive element could generate new ideas relating to the research. It was also suggested that, when forming smaller groups in meetings, the members should rotate and change to encourage a range of discussions across the groups.

### 5.10 Communication and Inclusivity

The communication from the project public involvement Lead was considered to work well and valued the clear and consistent communication across the project. The inclusive nature of the meetings and open discussions was valued, and members reported this was not always the case, as was the case in other public involvement meetings they had attended. The consistent offer of hybrid working allowed flexibility and made it possible for people to join if they were unable to attend meetings in person. Meetings were considered to be well organised, and the public contributors appreciated that they had an input on the locations of meetings and were given a choice in the dates and times of meetings.

For further improvements, it was suggested that a work plan and timeline of the public involvement activities over the entirety of the project would help people to understand the next steps and how their contributions would be embedded throughout the project.

There was a sense of disconnect during gaps in between meetings, and to remedy this, it was suggested that the researchers could provide updates between meetings on progress with the project. This communication would help the public contributors keep track of any new developments relating to the project.

It was also considered useful to have information about how the input from the group had made a difference to the research. This information would help the public contributors to know the difference they are making as the project progresses.

### 5.11 Diversity of Views and Representation

The group members valued being part of a diverse group of people who had a range of perspectives and backgrounds. They proposed that to learn more about one another, an online bio featured on the project webpage could be created for each group member (if they wish to do this) detailing their backgrounds, interests and experiences.

In conversations about future training, they suggested that training in how to take information about research back to communities and invite researchers to speak to communities would be a worthwhile investment. This training would equip the public contributors to bridge the gap between research and communities, meaning that the research produced is relevant to different communities that are currently under‐represented in research. In addition, to reach underserved communities, it was suggested that the research team should actively go into community settings to meet people and talk about research.

Recommendations made for the next steps regarding the project reported in this paper were on how outcomes of the project are tailored so they are accessible and reach different communities. The group members also noted a specific community that is not often involved in research, the Chinese community. One member reported that they have links to that community and invited the research team on this project to present their work to the group. They also suggested that for future projects, an advisory group of public contributors and professionals (who are not involved in the project but may work in settings relevant to the project topic area) were recruited as their perspective may be different from an individual who is living with a particular health condition. It was considered that these additional perspectives would contribute towards providing a more holistic view.

It was also suggested that the research team should invite their colleagues who worked in policy roles so they could hear first hand about the needs within different communities and different topics and issues discussed in relation to the uptake of interventions used to increase vaccination uptake. This could also provide avenues to explore ways to bridge the gap between the project outputs and their implementation in practice. The public contributors felt that this would be an effective strategy to ensure the research produces more impactful outcomes.

### 5.12 Summary

These reflections highlight both shared and differing perspectives among the public contributors. Collectively, the contributors valued the safe, inclusive environment, opportunities for learning, diverse group dynamics and clear communication, which were considered to enrich the discussions and brought a range of perspectives into the project.

The group also identified areas for improvement, which included broadening outreach to more communities, particularly to include members from communities who may be less represented in research and may be less inclined to get involved in research. They also highlighted the importance of offering consistent feedback as an area for improvement and for the research team to focus on moving forward. Additionally, contributors made recommendations for the team to consider incorporating more interactive elements, like role‐play, to make meetings more engaging.

An area of discussion where there were differences in opinion related to the length of the meeting times. The flexibility in choosing meeting locations and times was valued; however, some felt that 2 h was a sufficient amount of time for a meeting, while others would have preferred longer meetings.

## 6 Discussion

Our paper provides a detailed account of how public contributors were recruited, how public involvement was integrated into a systematic review project and how this involvement had mutual benefits for both the research and the public contributors.

Key factors that were considered to contribute to our success included mutual respect for different views, investing time in the relationship‐building process and getting to know the group.

The importance of relationship‐building in public involvement has been reported and discussed in the wider literature and guidance [25, 26, 27, 28]. Investing time in developing trusted relationships helps to create inclusive opportunities which, in turn, enhance the richness of perspectives brought to research. Over time, these relationships can lead to equal partnerships, promote shared power in decision‐making and encourage continued involvement throughout a project. Additionally, they provide valuable opportunities for shared learning and growth across the team.

For the public contributors involved in this project, we believe that the relationships built with individual group members provided strong foundations that allowed them to speak freely. As a result, contributors felt safe to challenge the views of the researchers and make recommendations to improve their experience in future meetings. Furthermore, there was a consistently high level of involvement—five out of six meetings had full attendance. The group was considered a welcoming space that embraced diversity and cultural differences. Discussions between the public contributors generated rich insights that helped shape the project's coding framework and analyses while also challenging people's perspectives.

We believe that the flexibility in our approach and providing space and time for developing relationships and recruiting a diverse group of public contributors greatly contributed to enhancing public involvement in the research project and was essential for ensuring meaningful involvement, as it created safe spaces for reflection and feedback and dynamic group discussions.

The importance of working with diverse groups is unmentioned in the current guidance for involving public contributors in systematic reviews [29, 30, 31].

Working with communities who are underserved in research is essential for ensuring that research is equitable and responsive and considers the needs and preferences of different communities [1, 32, 33, 34].

Reflecting on approach, we realise that it is possible that some valuable insights could have been missed if individuals were unable to fully express themselves in their preferred language, which could have created barriers to involvement. To address this, in future workshops we would ensure that if required interpreters were provided, materials were developed in multiple languages.

Furthermore, suggestions from the public contributors highlighted the importance of developing outreach plans to ensure that the findings are accessible and can be shared with wider communities who could benefit from the research.

We recommend that researchers allocate sufficient time and resources to make sure that a diverse group is recruited to shape systematic review projects. We would also suggest that plans be built into the project for disseminating research into communities and providing interpreters and materials in different languages.

Despite the recognition of the importance of public involvement in research to ensure its relevance, accessibility and acceptability, its integration within the systematic review process remains poorly understood. There is a lack of information about how public contributors are meaningfully involved in systematic reviews and subsequently how they may influence the research design and outcomes [35, 36].

As noted by Zhou et al. (2024), funding was a potential barrier in 11.8% of the reviews included. However, of the reviews that reported public involvement, 69% reported receiving funding, which is similar to the 67% of papers that did not report public involvement but did report receiving funding. This contradicts funding as a potential barrier because the percentage of papers receiving funding does not appear to differ significantly in regard to reporting public involvement. That stated, the funding itself may not have been for public involvement activities.

Zhou et al. also note an increase in public involvement activity reported in the papers from 5.9% in 2015, rising to 44% in 2022. There are several reasons why this may have been the case. It could be related to the development of training and resources around involving public contributors in systematic reviews [6, 29, 37] or linked to the requirement of most if not all major health and social care funders in the United Kingdom to include public involvement in research (31% of reviews included in Zhou et al. were from the United Kingdom), or due to the requirement from journals in addition to the BMJ such as Health Expectations and Research Involvement and Engagement to report public involvement activities, including those in systematic reviews [14, 29, 30, 37, 38, 39].

There are additional factors that may have influenced the inclusion and reporting of public involvement in systematic reviews [5, 14]. For example, the Covid‐19 pandemic changed working practices, including those related to public involvement [40, 41]. At the start of the pandemic, there was an initial decline in activity, but efforts were made to develop new approaches to public involvement, such as using online platforms [40, 42]. These changes created new ways of working, and in some cases, increased public involvement, which may have contributed to the observed rise in reporting. Other possible reasons were indicated during our reflective session with the public involvement group. During the discussion, the group members highlighted how this type of methodological research could initially be perceived as quite ‘dry’. We addressed this challenge by providing training on systematic reviews at the start of the project and breaking the sessions' structure into small, manageable and interactive discussion activities. After each activity, feedback was collected to understand how improvements could be made in real time, rather than at the end of the project. For example, group members expressed a preference for small group activities over whole group discussions, which influenced our approach to planning the sessions moving forward. It was also suggested that the use of an audible timer would help to ensure that we kept to timings as a group and were able to discuss a range of topic areas during our meetings. Furthermore, as a result of the open discussions, we also received new ideas, such as incorporating role‐play in the sessions to make them more engaging.

Another area identified for improvement was the need to provide feedback to the public contributors about the difference they had made to the project. The lack of such feedback has been cited as a recurring issue that can strain relationships between researchers and public contributors, as acknowledged by the work of Mathie et al. 2018 [43]. The UK Standards for Public Involvement, published in 2018, provide a benchmark of what good public involvement practice looks like and aim to improve the quality of public involvement practices [28]. There are six standards, and within each standard, indicators are provided to provide guidance on how that standard could be met. The standard ‘Communications’ indicators suggest that communication plans are developed for different activities, that the needs of different people are being met through inclusive communication methods, that processes are in place to offer, gather and share feedback, and that public involvement learnings and achievements are being shared including the good and the bad [28].

Mathie et al. published a study that aimed to improve the feedback experience for public contributors [44]. The study highlighted the essential role of public involvement leads in facilitating feedback between researchers and the public and emphasised the necessity of ensuring that there are sufficient resources and support for this role. It was also highlighted that there were great benefits to creating bespoke feedback mechanisms that are developed jointly between the public contributors and project team [44].

As a result of the learnings gained through our project, we recommend that researchers develop a communication and feedback plan with public contributors from the start of the project. We also suggest that a public involvement lead role be budgeted for, with part of their responsibilities focused on overseeing and developing these feedback processes [28, 43]. By doing so, researchers can ensure effective strategies and regular opportunities for feedback are embedded throughout the project.

In relation to how we will address this area in our project, the public contributors have been involved in writing this paper, with two public contributors as co‐authors. Following our discussions with the group, we have also planned a session to provide an overview of the project progress and outline the impact of the public involvement and will repeat this at the end of the project.

The ACTIVE framework is a helpful structure for reporting our involvement [6]. Reflecting on our approach to involvement using this tool has enabled the team to recognise that within each stage of the systematic review process outlined on the framework, there can be multiple steps within one stage that enable patients and the public to be involved meaningfully and shape the review process. We would recommend to others as a practical step that this framework could provide a useful tool as a starting point for discussions about how public contributors can be involved in systematic reviews and as a framework for reporting public involvement activities.

Our next steps will involve building on our current practices and using the feedback and recommendations from the group to shape our future meetings and activities. We will work together to interpret the project findings and develop ways to effectively disseminate information about the research to wider communities (Table 3).

**Table 3:** Key recommendations for involving public contributors in systematic review projects.

Key recommendations
1. Invest time in building long‐term relationships and trust with public contributors and community organisations to enhance public involvement. 2. Ensure there are resources available to support public contributors' individual learning needs, which may include training related to systematic reviews. 3. Ensure that findings and next steps are shared with public contributors to ensure they understand the impact of their collective efforts. 4. Be flexible when designing meeting structures, which should be developed based on input and feedback from the public contributors. Consider using creative activities such as role‐play, where possible, to actively engage people in the research. 5. Offer pre‐meetings and support to provide opportunities for public contributors to ask questions and contribute to the meeting formats. 6. Develop an inclusive outreach plan and dissemination strategy to ensure that information about the research is shared in ways that are accessible. This includes using appropriate language when reaching out to different communities. 7. Ensure your public involvement group reflects the diversity of people affected by your research. 8. Make sure you have allocated sufficient funds within your budget for public involvement throughout the research process. This includes the cost of public contributors' time and including other costs such as for the provision of training and relationship‐building.

## 7 Conclusion

This paper adds to research that has already been undertaken to report and develop effective approaches for involving public contributors in the systematic review process. We have reported how public contributors have been involved and offered recommendations based on our experiences to support others in developing their own approaches to effective public involvement in systematic reviews.

## Author Contributions

C.M. produced the first draft of the manuscript. All authors contributed to drafting and editing the article and approved the final manuscript.

## Disclosure

The views expressed are those of the authors and not necessarily those of the NIHR or the Department of Health and Social Care.

## Ethics Statement

As per the Health Research Authority/NIHR INVOLVE statement, ethical approval was not required for this patient and public involvement piece [45].

## Consent

Written and verbal consent was provided by all public contributors to publish the content included in this paper.

## Conflicts of Interest

The authors declare no conflicts of interest.

## Supporting information

Supmat.

## Data Availability

All data generated or analysed during this study are included in this published article.
