# Supplementary material for: Public Involvement in a Systematic Review Project: Reporting Our Approach Using the ACTIVE Framework
Source: Health Expect. 2025 Jun 30;28(4):e70323. doi: 10.1111/hex.70323 (PMC12218336; doi:10.1111/hex.70323)
Supplement: Supplementary file 1 — Supmat. [file HEX-28-e70323-s001.docx]

## Appendices

Appendix 1: Self- reported demographic information of public contributors involved *

| **Description** | **All Respondents (n=7)** |
| --- | --- |
| **Age** |  |
| Prefer not to say |  |
| 19 years or younger |  |
| 20-24 |  |
| 25-29 | 1 |
| 30-34 |  |
| 35-39 | 2 |
| 40-44 | 1 |
| 45-49 | 1 |
| 50+ | 4 |
| **What is your ethnic origin?** |  |
| Prefer not to say |  |
| Arabic |  |
| Middle East |  |
| Asian or Asian British- Bangladeshi |  |
| Asian or Asian British- Chinese | 1 |
| Asian or Asian British- Indian | 1 |
| Asian or Asian British- Pakistani |  |
| Asian or Asian British- Any other Asian* |  |
| Black or Black British-African | 3 |
| Black or Black British-Caribbean | 1 |
| Black or Black British-Any other Black background* | 2 |
| Mixed- White & Asian |  |
| Mixed- White & Black African |  |
| Mixed- White & Black Caribbean Background |  |
| Mixed- any other mixed background* |  |
| White- British | 1 |
| White-European |  |
| White- Mediterranean |  |
| White- any other white background* |  |
| Other ethnic group- any other ethnic group* |  |
| *If your ethnic origin is not listed, or you selected any ‘other’ category, please describe. |  |
| **What is your general attitude towards being vaccinated?** |  |
| Happy to receive recommended vaccines | 3 |
| Some hesitation / not quite sure / it depends on the vaccine | Some hesitation x3  Depends on the vaccine x1 |
| I’m not keen to be vaccinated | 1 |
| **What is your employment status? (e.g. full time; part time; unemployed; carer)** |  |
|  | Early retired x1  Part time x1  Full-time x1  Health Retirement x1  Unemployed- Long term illness x1  Semi-retired x1  Unemployed x1 |
| **Do you consider yourself to have a disability?** |  |
| Prefer not to say |  |
| No | 6 |
| Yes If you wish to expand, please identify the category or categories which apply to you: | 3 |
| Physical impairment | 2 |
| Long-standing illness | 3 |
| Sensory Impairment | 2 |
| Learning disability/difficulty |  |
| Mental health condition |  |
| Other | Genetic illness |
| **What is your highest level of completed education?** |  |
| Prefer not to say |  |
| No formal education |  |
| Primary School |  |
| Secondary School | 1 |
| Sixth form or college | 2 |
| Technical Education |  |
| Undergraduate study (eg Bachelors) | 4 |
| Postgraduate study (eg Masters, PhD) | 2 |
| Other |  |
| **Do you belong to any particular religious or faith group?** |  |
| Prefer not to say | 1 |
| No | 1 |
| Yes, if you would like to share which religion or faith group you belong to, please do: | Yes  Catholic x 2  Christian x3  Hindu |

* This was a self-reported survey therefore not all group members have responded to all (or any) of the questions.

Appendix 2: Key considerations


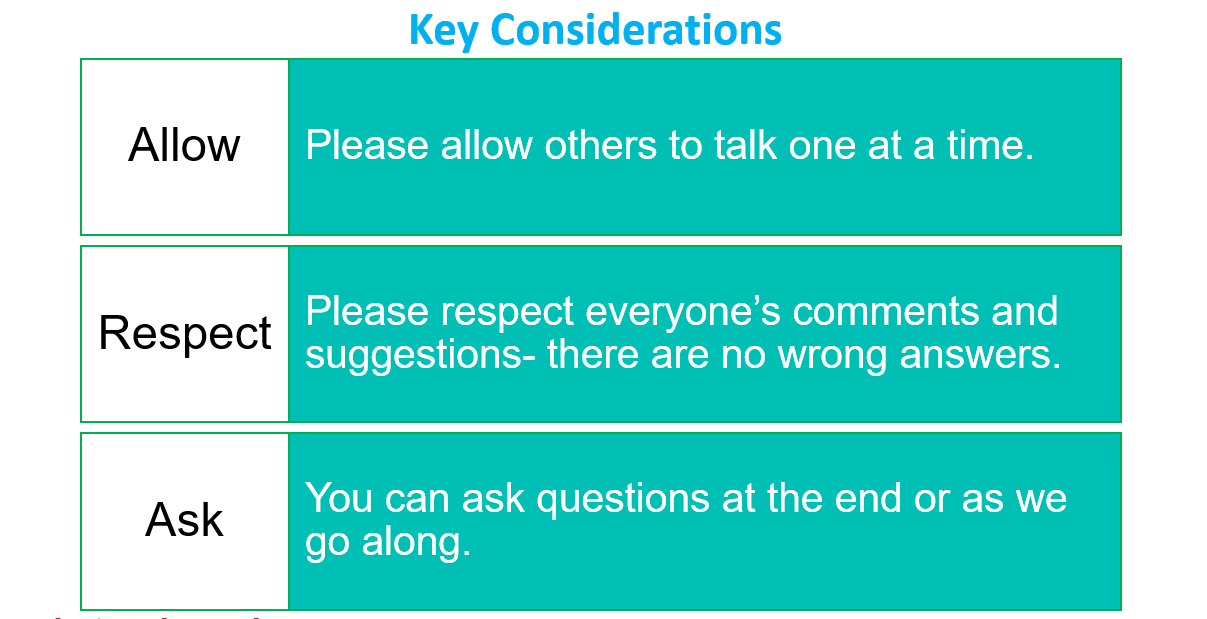


## Appendix 3: Map of the world used for our first public involvement meeting


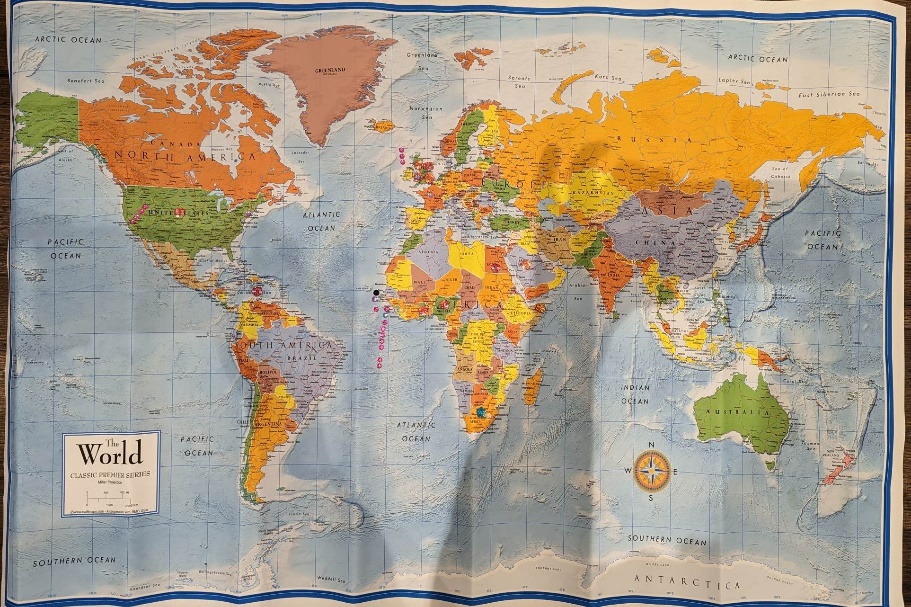


Appendix 4: Role descriptor created for the public involvement group members

| **Vaccine Uptake review Public Involvement- Role descriptor.** |
| --- |

| **What is the project?**  Vaccination is very good at reducing illness and death from infectious diseases. However, for vaccination programmes to work well it is important that most people get vaccinated. This protects their own health and limits the amount of disease in the population. When not enough people are vaccinated, diseases can spread quickly.  **What is the problem?**  Making sure most people take up the offer of vaccination is difficult. For example, the number of children getting routine vaccinations in England has gone down in recent years, meaning more children are catching potentially serious diseases.  We have also seen that the number of people getting COVID-19 vaccines is lower in some communities. The number of people getting vaccinated in general is lower in some populations, including some ethnic minority groups and deprived communities. As a result, these communities are experiencing poorer health.  Our research will help find the best ways to support these groups to get vaccinated. We will search for and review studies reporting on strategies to increase the uptake of vaccines, including studies on all population groups and vaccines. We will also consider the costs of the strategies that we find to work best.  We want to work with you to help us make shape our research is going to be relevant and useful for people. We also want to offer you opportunities for learning and developing new skills.  **What is the broad purpose / role of the public involvement group?**  As a member of this project, you will be a public partner working closely with myself (Carmel), and a group of eight other public members. We will also be working with Dr. Clare French who is leading this project and Dr. Sarah Davies who is a researcher on this project. We will be working together at each stage of the project. We will ask you to share your thoughts and ideas and help us to develop parts of the project. We will provide training, and we will ask you to tell us what type of training you would be interested in.  **What are the aims / responsibilities of the group?**  We will work together on this project taking a collaborative approach to public involvement. The definition states that [collaboration](https://www.invo.org.uk/resource-centre/jargon-buster/) as:  “An on-going partnership with members of the public in the research process. For example, members of the public might take part in an advisory group for a research project, or collaborate with researchers to design, undertake and/or disseminate the results of a research project”.  At times, there may be parts of the project that we cannot change. However, by working closely together in this way we will be open and transparent about these decisions, and we will identify areas of the project where it is possible to change.  **What will you be expected to contribute to the group in broad terms?**  We expect you to:   - Contribute your own honest interpretations and opinions of our findings. - Share your experiences and insights - Discuss your views and experiences around vaccination - Be committed to the role and support the research team where possible. |
| --- |

| **Your responsibilities** |
| --- |
| The most important aim of involving you in this project is to make sure that it is an enjoyable, learning experience. Therefore, we ask that you:   - Ask questions throughout - Provide your opinions and suggestions freely - Are willing to learn new skills. - Let us know how we can support you. - Be open and transparent about all aspects of the project (i.e. if you can no longer be involved if you are finding the process difficult). |

| **Our responsibilities** |
| --- |
| Throughout this project it is our responsibility to:   - Support you as much as required. - Provide you with enough information and time to complete the tasks. - Be open and transparent about where you will be able to make a difference to the research. - Be responsive to your suggestions and provide feedback relating to the research and aspects of the project (i.e., when we have meetings, when you are paid). - Provide you with additional contacts/ support. - Encourage and help you to learn new skills. - Provide regular feedback |

| **Duration of the role** | |
| --- | --- |
| The project will be running over two years. We would like to meet with you every four months. In total, this means we would like you to come to a **minimum of six meetings.** There may be times when we organise more meetings, for example, to provide you with some additional training.  Please see below for a rough schedule of planned activities and meeting dates although this may change according to the needs of the project. Meeting dates can also be changed based on your availability. | |
| **When is the Activity?** | **How long is the activity?** |
| November 2022 | 2 hours |
| March 2023 | 1.5 hours |
| July 2023 | 1.5 hours |
| November 2023 | 1.5 hours |
| March 2023 | 1.5 hours |
| July 2023 | 1.5 hours |

| **Payment and expenses** |
| --- |

| **How much will you be paid per hour?**  We will reimburse your time paying you £25 per hour as per the [National Institute for Health and Care (NIHR) payment guidance](https://www.nihr.ac.uk/documents/payment-guidance-for-researchers-and-professionals/27392).  We will pay you for your time preparing for meetings (e.g. if there are papers to read before the meeting).  We can also reimburse you for your travel to and from the meetings.  **How will you be paid?**  We can pay you with Love2Shop vouchers or a direct bank transfer. You can let us know which option works best for you.  **Additional information you need to know.**  For more information about the NIHR payment guidance please follow this link: [Payment guidance for members of the public considering involvement in research \| NIHR](https://www.nihr.ac.uk/documents/payment-guidance-for-members-of-the-public-considering-involvement-in-research/27372). Within this guidance there is useful information about reimbursement for public involvement if you are receiving benefits. |
| --- |

Appendix 5: A table showing how the three different public involvement group members grouped and categorised the different intervention components.

|  | **Group 1** | **Group 2** | **Group 3** |
| --- | --- | --- | --- |
| **Who delivered the intervention ?** | **Category 1:** Community Advisors or Trusted People or Trusted Community Members | **Category 1:** Trusted community based | **Category 1:** Community led |
|  | Teachers  Social workers  Religious leaders  Community leaders  Peers  Other | Religious leaders  Community leaders  Health visitors  Peers | Peers  Community leaders  Social workers  Teachers  Researchers  Religious leaders  Other (depending on what it was) |
|  | **Category 2:** Healthcare Professional or Health Advisors | **Category 2:** Medical | **Category 2:** Health practitioner |
|  | Researchers  Health visitors  Nurse  GP  Midwives  Pharmacists  Practitioners  (Note - the group were happy to group ‘researchers’ with other health professionals since people will assume that a researcher working on vaccination/providing information about vaccination will have some medical knowledge) | Practitioners  GP  Pharmacist  Nurse  Social worker  Teacher  Midwives | Practitioners  GP  Pharmacist  Nurse  Midwives  Health visitor  Other (depending on what it was) |
